# Infiltrative pattern of metastatic invasive lobular breast carcinoma in the abdomen: a pictorial review

**DOI:** 10.1186/s13244-021-01120-4

**Published:** 2021-12-11

**Authors:** Ying Mei Wong, Pooja Jagmohan, Yong Geng Goh, Thomas Choudary Putti, Samuel Guan Wei Ow, Yee Liang Thian, Premilla Pillay

**Affiliations:** 1 Department of Diagnostic Imaging, National University Hospital, Singapore, 1E Kent Ridge Road, NUHS Tower Block Level 12, Singapore, 119228 Singapore; 2 Department of Pathology, National University of Singapore, National University Hospital, Kent Ridge Road, Singapore, 119074 Singapore; 3 Department of Hematology-Oncology, National University Cancer Institute and Yong Loo Lin School of Medicine, National University of Singapore, 1E Kent Ridge Road, NUHS Tower Block Level 7, Singapore, 119228 Singapore

**Keywords:** Carcinoma (lobular), Breast, Abdominal cavity, Tomography (X-ray computed), Positron emission tomography computed tomography

## Abstract

Invasive lobular carcinoma (ILC) has a greater tendency to metastasize to the peritoneum, retroperitoneum, and gastrointestinal (GI) tract as compared to invasive carcinoma of no special type (NST). Like primary ILC in the breast, ILC metastases are frequently infiltrative and hypometabolic, rather than mass forming and hypermetabolic in nature. This renders them difficult to detect on conventional and metabolic imaging studies. As a result, intra-abdominal ILC metastases are often detected late, with patients presenting with clinical complications such as liver failure, hydronephrosis, or bowel obstruction. In patients with known history of ILC, certain imaging features are very suggestive of infiltrative metastatic ILC. These include retroperitoneal or peritoneal nodularity and linitis plastica appearance of the bowel. Recognition of linitis plastica on imaging should prompt deep or repeat biopsies. In this pictorial review, the authors aim to familiarize readers with imaging features and pitfalls for evaluation of intra-abdominal metastatic ILC. Awareness of these will allow the radiologist to assess these patients with a high index of suspicion and aid detection of metastatic disease. Also, this can direct histopathology and immunohistochemical staining to obtain the correct diagnosis in suspected metastatic disease.

## Key points

- Compared with NST, metastatic ILC in the abdomen more commonly involves the peritoneum, retroperitoneum, and gastrointestinal tract.
- ILC metastases are frequently infiltrative, rendering them challenging to identify on imaging.
- When a discrete mass is not seen on imaging, presence of infiltrative metastases is sometimes more readily inferred by secondary complications like bowel obstruction
- Recognition of linitis plastica of the bowel on imaging should prompt deep or repeat biopsies.
- Awareness of these imaging pitfalls can aid detection of metastatic disease on imaging.

## Background

Invasive lobular carcinoma (ILC) is the second most common histological type of invasive carcinoma after invasive carcinoma of no special type (NST), which was previously known as invasive ductal carcinoma (IDC) [1, 2]. ILC accounts for 5–15% of all breast cancer cases [3, 4].

Common sites of metastases for both ILC and NST include the bones, liver, lungs, and non-axillary lymph nodes [5–9]. However, ILC has a greater tendency to metastasize to the peritoneum, retroperitoneum, and gastrointestinal (GI) tract as compared to NST. ILC metastases are often infiltrative and subtle, rendering them difficult to detect on imaging as compared to mass-forming lesions. Clinical stage of ILC has been shown to be higher than NST at presentation and patients with abdominal ILC metastases have a shorter overall survival [8, 10].

Metastatic ILC can present with late relapse many years after remission. GI metastases can be seen as late as 15 years after initial diagnosis [11]. Consequently, patients may even fail to declare their history of breast cancer due to a prolonged disease-free interval [12, 13].

In this pictorial review, the authors aim to familiarize readers with the infiltrative pattern of metastatic spread of ILC in intra-abdominal sites. This should raise the index of suspicion of radiologists when imaging patients with a history of ILC. As imaging findings could be subtle and easily missed until the disease is extensive with clinical manifestations, the authors would like to emphasize the importance of discussion with clinicians for ILC patients at various stages of disease (i.e. early stage, remission, or late relapse). There should be a low threshold for imaging when patients present with abdominal signs or symptoms.

## Pathology of ILC

The distinct molecular and histopathologic features of ILC and NST account for their different manifestations and sites of metastases [14–16]. The infiltrative pattern of growth of ILC is the result of loss of E-cadherin, the cell-to-cell adhesion molecule, which is related to changes at the genomic level [17].

**Fig. 1:**
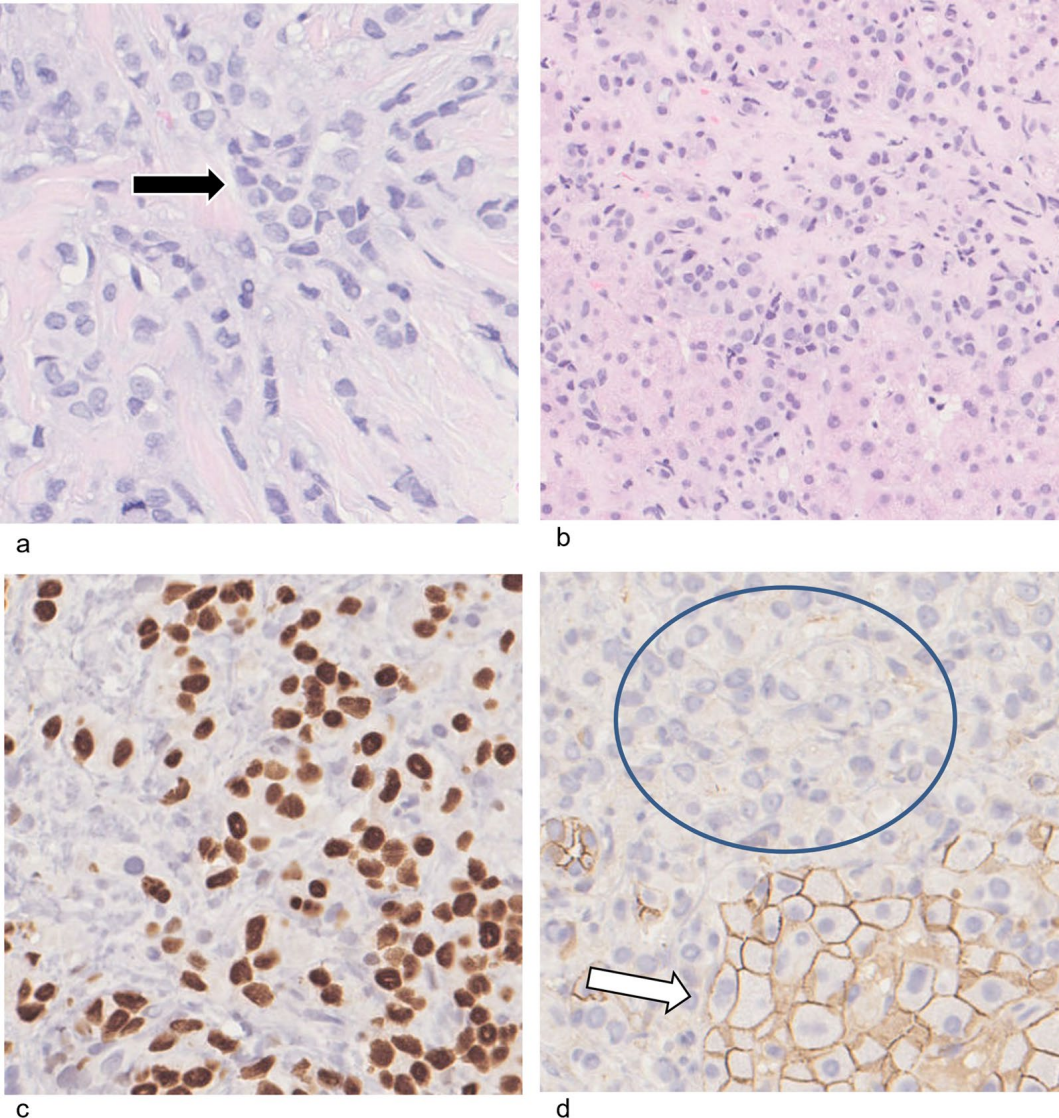
Pathology and immunohistochemical staining of invasive lobular carcinoma. **a** In the breast: high power photomicrograph (H&E) shows the single-file appearance of primary ILC in the breast (black arrow). **b** In the liver: low power photomicrograph (H&E) demonstrates tumor cells infiltrating the liver. **c** In the liver: positive staining for GATA3 antibody suggests origin from the breast. **d** In the liver: negative E-cadherin staining of ILC cells (circled) and positively stained normal liver cells (white arrow)

On histology, primary ILC is characterized by small round cells which infiltrate the breast stroma in single-file (“Indian file”) pattern (Fig. 1a) [18]. They encircle benign mammary ducts in a targetoid fashion and do not destroy anatomic structures or incite substantial connective tissue response [3]. The majority of ILC is hormone receptor positive, HER2-negative, and luminal subtype A [18].

Metastatic ILC can have a similar single-file appearance on histology, although this can be difficult to differentiate from other metastases or from a primary tumor of the organ in question. Immunohistochemistry is necessary to clinch the diagnosis. Positive staining with GATA3 may indicate a breast primary, while negative staining for E-cadherin strongly suggests ILC (Fig. 1b–d) [19, 20].

## Imaging of primary ILC of the breast

**Fig. 2:**
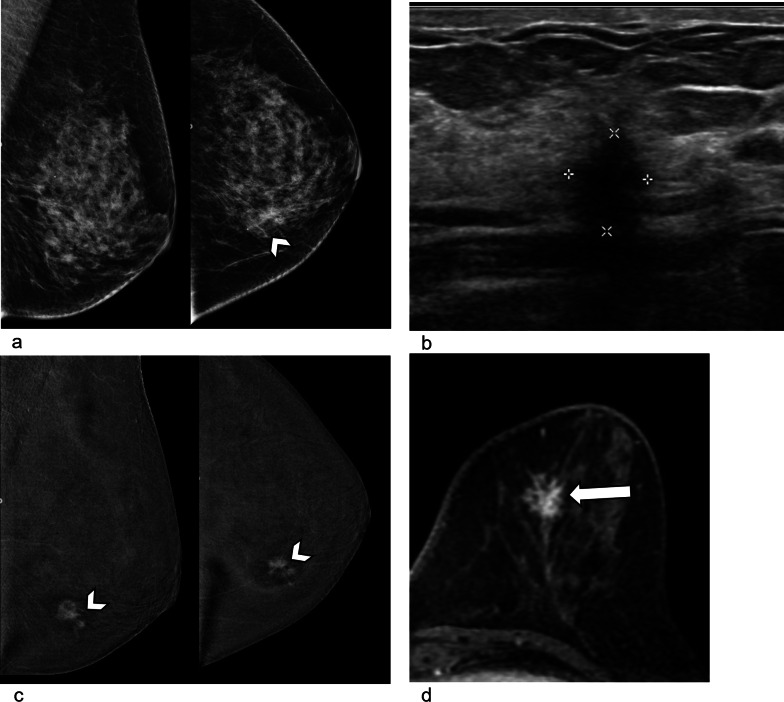
Primary ILC of the breast in a 61-year-old female who presented with a left breast lump. **a** Mammogram of the left breast shows focal asymmetry in the inner left breast on CC view (arrowhead), which is not well seen on MLO view. This corresponds to the palpable lump. **b** Ultrasound of the lower inner left breast demonstrates a corresponding irregular hypoechoic 1.0 cm nodule with posterior acoustic shadowing. This was proven on biopsy to be invasive lobular carcinoma. **c** Subtracted CEDM of the left breast shows the known ILC is enhancing with spiculated margins (arrowheads), with a tiny adjacent nodule not seen on ultrasound. The tumor measured larger on CEDM (1.6 cm) than on ultrasound (1.0 cm). **d** Axial contrast-enhanced MRI image of the left breast shows the spiculated, enhancing ILC (arrow), similar in size compared with CEDM. No other suspicious nodules were seen in either breast on CEDM and MRI

Due to its infiltrative pattern of growth, primary breast ILC is often challenging to diagnose on imaging. On mammography, it can manifest as a mass with ill-defined/spiculated margins, focal asymmetry (Fig. 2a), or architectural distortion [21, 22]. Digital breast tomosynthesis (DBT) has been shown to improve the detection of ILC manifesting as asymmetric densities and distortions [23]. On ultrasound, ILC typically appears as an irregular, hypoechoic mass, or ill-defined area of hypoechoic change with posterior acoustic shadowing (Fig. 2b) [7, 21].

Contrast-enhanced studies like magnetic resonance imaging (MRI) and contrast-enhanced digital mammography (CEDM) visualize the enhancing neovascularity of tumors and greatly improve assessment of tumor extent (Fig. 2c, d) and delineation of non-mass enhancement [24]. Contrast-enhanced studies also improve detection of unsuspected multifocal, multicentric, and bilateral disease, which is important for surgical planning [7, 25].

Primary ILC demonstrates lower standardized uptake values (SUV) and is less appreciable than primary NST on ^18^F-FDG PET/CT [15, 26].

## Imaging of metastatic ILC in the abdomen

Like ILC in the breast, early metastatic ILC tends to be infiltrative, rather than mass forming.

^18^F-FDG PET/CT is less sensitive for staging of ILC than NST. PET/CT is less likely to reveal unsuspected distant ILC metastases, and if patients are upstaged based on CT findings, these are often not ^18^F-FDG-avid [27].

In the following sections, this infiltrative appearance of metastatic ILC in the abdomen on imaging and its mimics will be discussed. Incidences comparing ILC and IDC have been illustrated in several autopsy and imaging series [5, 6, 8, 10] and these will be discussed in the organ specific sections below.

### Metastatic ILC in the liver

**Fig. 3:**
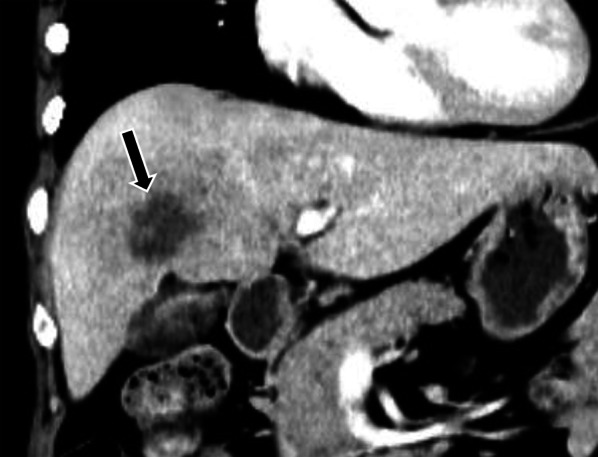
Typical mass-forming appearance of metastatic ILC in the liver in a 62-year-old female. She had Stage 1B ILC treated 4 years ago and was on adjuvant exemestane. Coronal contrast-enhanced CT image in the liver window shows a dominant hypodense mass in the liver (arrow) along with multiple other hepatic nodules (not pictured). Percutaneous biopsy of the liver mass showed metastatic ILC

The liver is one of the most common sites of metastatic ILC, with autopsy series showing involvement in 43–68% of cases [5, 6]. Prior studies have demonstrated similar incidence of hepatic metastases in both ILC and NST [10, 28]. In their series of 57 patients with metastatic ILC, Winston et al. identified two patterns of metastatic spread in the liver [29]. The first pattern is the frequently reported appearance of discrete hepatic masses, which are indistinguishable from other metastases (Fig. 3) [9]. The second type of spread is an infiltrative pattern with distortion of hepatic vessels. The proportion of each pattern of spread was, however, not reported in their study.

**Fig. 4:**
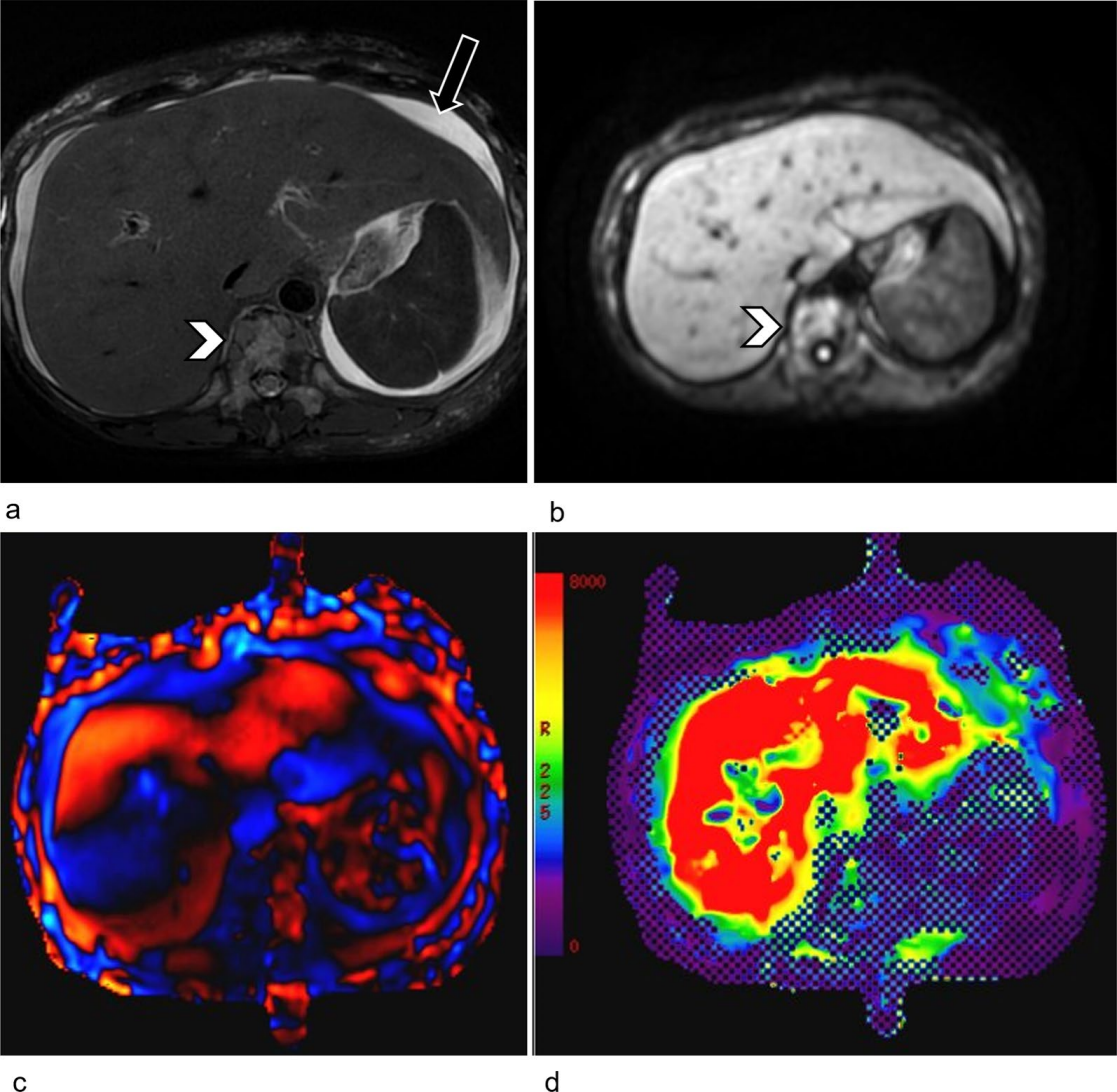
Metastatic ILC of the liver mimicking hepatic fibrosis in a 55-year-old female who presented with jaundice. She had a remote history of Stage 3C ILC more than 10 years ago. **a** Axial, fat-saturated, T2-weighted MR image and **b** diffusion-weighted image (b = 500 s/mm^2^) do not show any discrete hepatic lesion. The hepatic outline is smooth, with no morphological features of cirrhosis. Ascites is present (arrow). Bone metastases are also noted (arrowheads). **c** Wave images from the MR elastography acquisition at 60 Hz show shear waves with prolonged wavelength. **d** MR elastogram demonstrates severely increased mean shear stiffness in the liver, with corresponding quantitative color scale on the left of the image. Mean stiffness value is 15.6 kPa (normal < 2.93 kPa), in keeping with F4 fibrosis. The diagnosis of infiltrative metastatic ILC was confirmed on percutaneous liver biopsy

In this section, we emphasize the second infiltrative pattern which is not commonly reported in the literature. In this pattern, the liver can retain a smooth outline, with no morphological features of cirrhosis (Fig. 4a, b). Hence, the diagnosis is often missed in the early stage and patients would present much later with biochemical and clinical evidence of liver failure (e.g. jaundice or non-malignant ascites) [30, 31]. As the underlying infiltrative disease is not apparent on conventional imaging modalities, such as ultrasound or CT, the authors would recommend further imaging with MRI elastography (MRE) or ultrasound-based elastography (Fibroscan) to quantify liver stiffness as a surrogate for infiltrative disease. In the context of relatively normal appearance of the liver on conventional imaging and absence of typical risk factors for developing cirrhosis, severely elevated liver stiffness (Fig. 4c, d) in a patient with history of ILC should raise the suspicion of infiltrative hepatic ILC metastases. Familiarity with this second pattern of infiltrative spread in the liver can direct immunohistochemical stains to clinch the diagnosis.

### Metastatic ILC in the pancreas

Pancreatic metastasis from breast cancer is uncommon, with the majority of the literature consisting of case reports [32–34]. Case series by Winston et al. and Switzer et al. showed an incidence of 2–5% metastatic ILC involvement of the pancreas [29, 35]. Metastatic ILC in the pancreas often presents as a discrete mass, which is indistinguishable from primary pancreatic tumors on imaging.

**Fig. 5:**
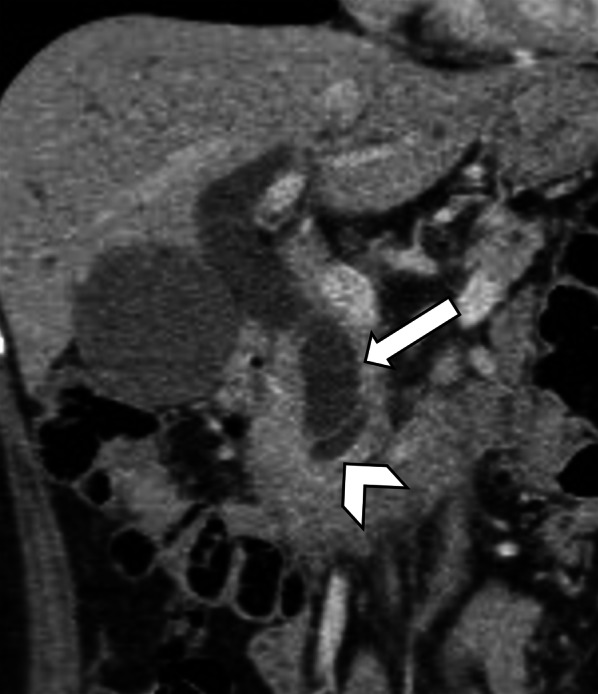
Metastatic ILC of the pancreas in a 53-year-old female presenting with painless jaundice. She was on adjuvant letrozole for Stage 2 ILC of the left breast diagnosed 2 years ago. Coronal contrast-enhanced CT image shows a dilated common bile duct (white arrow) and dilated pancreatic duct (arrowhead)—the double duct sign. The intrahepatic ducts are also mildly dilated. No discrete mass is seen in the pancreatic head on CT or on EUS. Metastatic involvement of the pancreatic head was confirmed on intra-operative biopsy

Like its infiltrative pattern in other parts of the body, ILC metastases in the pancreas can be subtle on imaging. A discrete mass may not be clearly discerned on cross-sectional imaging or endoscopic ultrasound (EUS). Instead, the presence of metastatic infiltration of the pancreas may be suspected in the context of painless jaundice and upstream duct dilatation on imaging. If the metastatic disease involves the pancreatic head, this may manifest with the “double duct sign,” in which the common bile duct and pancreatic duct are dilated (Fig. 5). Biopsy would be needed to distinguish this from a primary pancreatic head adenocarcinoma.

### Metastatic ILC in the gastrointestinal tract

Metastatic spread to the GI tract is more common in ILC than in NST. An autopsy series of ILC cases showed the incidence of GI metastases to be as high as 40%, compared with 2% in NST, although these usually remain clinically occult [5]. Studies have shown varying incidence of stomach, small bowel, and colon involvement [8, 9, 29]. Metastatic GI tract involvement is often multifocal [8, 35].

**Fig. 6:**
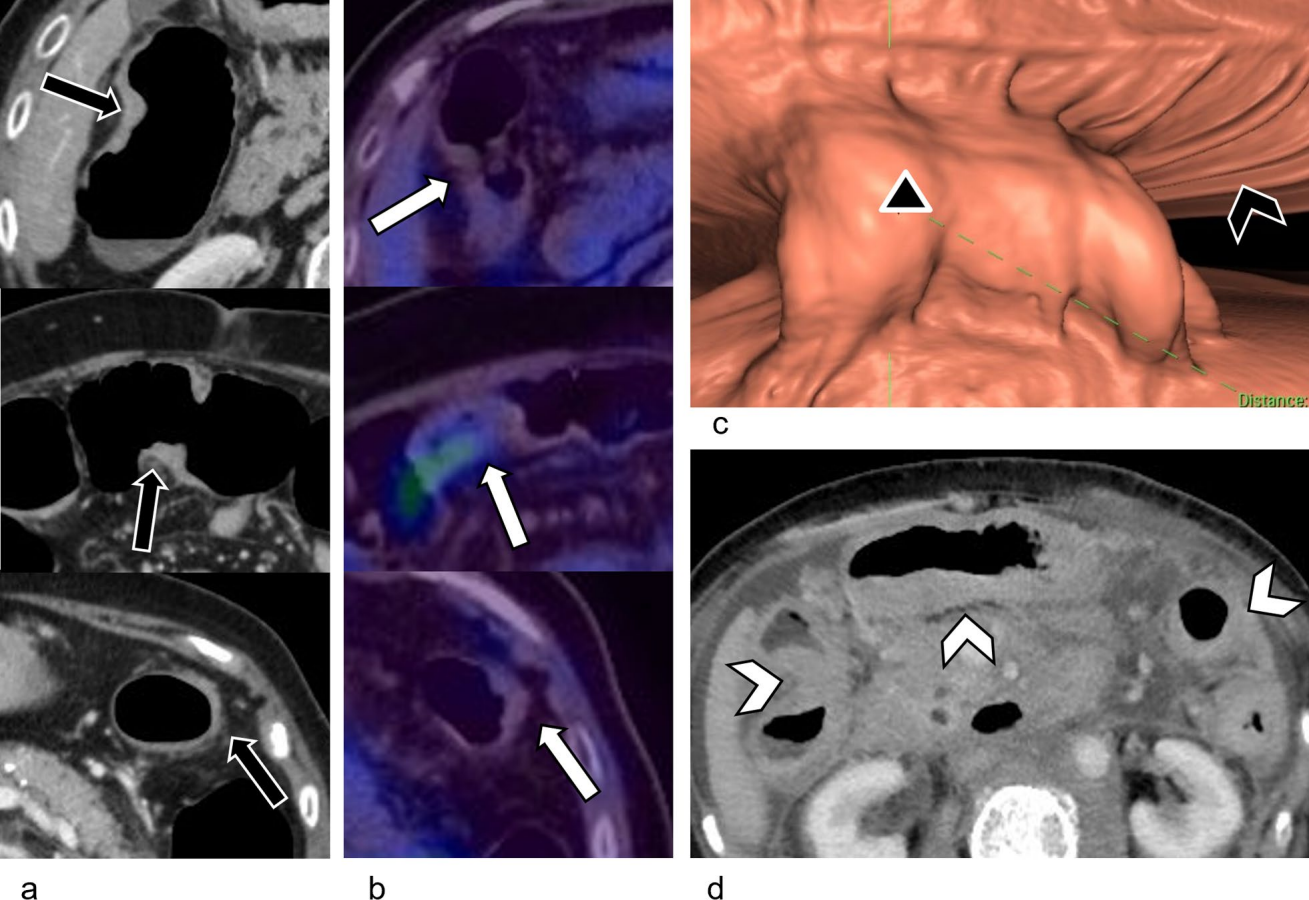
Metastatic ILC of the colon in a 77-year-old female with recurrent episodes of subacute intestinal obstruction over 6 months. She had completed therapy for Stage 3A ILC more than 10 years ago. **a** Axial contrast-enhanced CT colonography shows focal segments of smooth, mural thickening (black arrows) in the hepatic flexure (top), transverse colon (middle), and descending colon (bottom), mimicking peristalsis. No discrete mass is seen. **b** ^18^F-FDG PET/CT scan performed shortly after demonstrates that these segments of mural thickening in the colon (white arrows) have minimal ^18^F-FDG-uptake. **c** Reconstructed CT colonography virtual dissection image shows that the normal haustra of the hepatic flexure (black arrowhead) is disrupted by abnormal, focal mural thickening (∆), confirming that it is pathological. **d** Axial contrast-enhanced CT 1 year later shows progression of metastatic disease involving the hepatic flexure, transverse colon, and descending colon (white arrowheads)

Metastatic ILC in the GI tract is a diffuse spreading process, with the submucosa involved first [6]. Tumor infiltration appears as smooth bowel wall thickening on CT (Fig. 6a, d) [29]. This is challenging to recognize as pathological, particularly in the early stages, as it mimics peristalsis on CT and has ^18^F-FDG-avidity similar to normal physiological activity (Fig. 6b). However, 3D virtual dissection reconstruction derived from CT colonography can be helpful in differentiating between pathological thickening and peristalsis by demonstrating the disruption of the colonic haustra in the thickened segments (Fig. 6c).

**Fig. 7:**
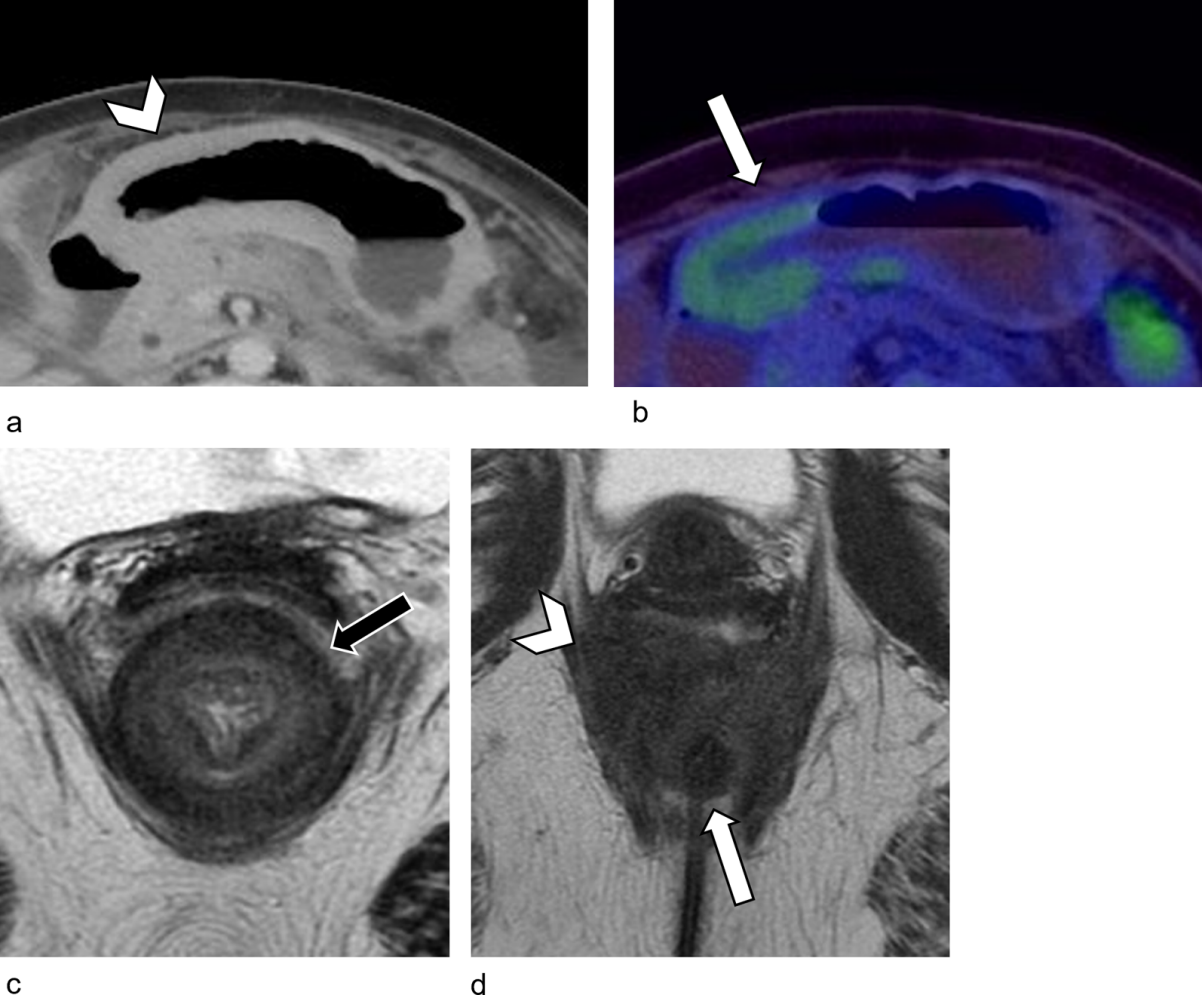
Linitis plastica appearance of the stomach and rectum due to metastatic ILC in a 61-year-old female. Six years after completing therapy for Stage 1A ILC of the right breast, she presented with change in bowel habit and a palpable rectal mass on examination. She later developed symptoms of subacute bowel obstruction. **a** Axial contrast-enhanced CT image shows incidental diffuse, circumferential wall thickening of the stomach (white arrowhead), suggestive of linitis plastica. **b** Corresponding axial PET/CT demonstrates mild ^18^F-FDG-uptake (SUVmax 3.05) in the distal stomach (white arrow). This degree of metabolic uptake was deemed at the upper limit of physiological uptake. Diffuse metastatic involvement of the stomach was confirmed on exploratory laparotomy. **c** Axial T2-weighted MR image demonstrates circumferential thickening of the rectum, with preserved concentric bowel wall layers (black arrow), giving a “target sign” appearance. **d** Axial T2-weighted MR image of the pelvic floor shows a near-circumferential, ill-defined, T2-weighted hypointense mass (white arrowhead) around the anal canal (white arrow). Initial biopsies were inconclusive, likely due to the predominantly extramural location of the mass. Metastatic involvement was confirmed on subsequent deep biopsy

When metastatic disease progresses and involves all layers of the stomach, it appears as linitis plastica [9, 13], in which the stomach is poorly distensible and diffusely thickened (Fig. 7a, b). This is the most common manifestation of ILC metastases in the stomach and mimics primary scirrhous gastric carcinoma on imaging [36]. Peritoneal disease is often associated with linitis plastica of the stomach [6].

Linitis plastica of the colon and rectum has also been described [37]. The bowel wall layers can also be preserved but diffusely infiltrated, giving a concentric ring or “target sign” appearance on imaging (Fig. 7c). Colorectal linitis plastica is usually secondary to primary malignancy in the stomach, prostate, and breast. Primary colorectal linitis plastica is rare [38]. These are identical on imaging and are only differentiated following biopsy.

Given that metastatic ILC is primarily submucosal, endoscopy may be falsely reassuring as the mucosa appears normal in the early stages. Even when mass forming, the tumor can be predominantly extramural, with minimal mucosal involvement (Fig. 7d). Initial/superficial biopsies can be normal in 46–50% of cases [39]. A low threshold to perform deep or repeat biopsies is needed if metastatic disease is suspected [13].

### Metastatic ILC in the peritoneum and retroperitoneum

ILC has a higher tendency to metastasize to the peritoneum and retroperitoneum compared with NST. Autopsy series of ILC patients have shown peritoneal metastases rates as high as 60–90% compared to 15% in NST [5, 6]. The ILC peritoneal metastases in these autopsy series were diffuse, in contrast to NST metastases which were nodular. Peritoneal disease is associated with poor prognosis [10].

**Fig. 8:**
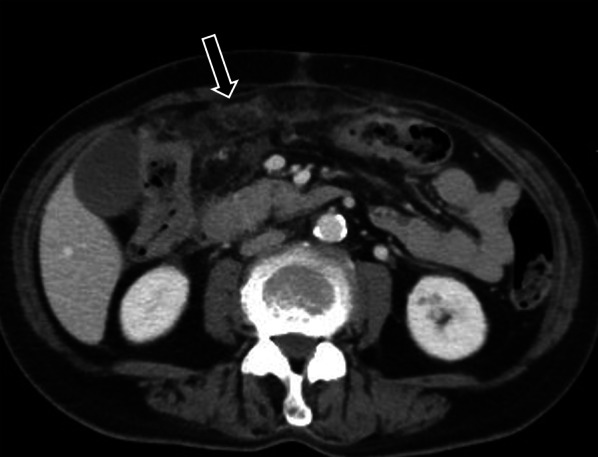
Metastatic peritoneal disease on staging CT, in a 61-year-old female with invasive lobular carcinoma. Axial contrast-enhanced CT image shows multiple, ill-defined, tiny nodules in the peritoneum (arrow), which were highly suspicious for metastatic disease. This was confirmed on diagnostic laparoscopy and biopsy

Early ILC metastases in the peritoneum and retroperitoneum are difficult to detect as they are clinically occult and manifest as tiny, indistinct nodules and stranding on imaging (Fig. 8). Knowledge of a history of ILC in the patient will alert the radiologist to assess this stranding and nodularity with a higher index of suspicion.

**Fig. 9:**
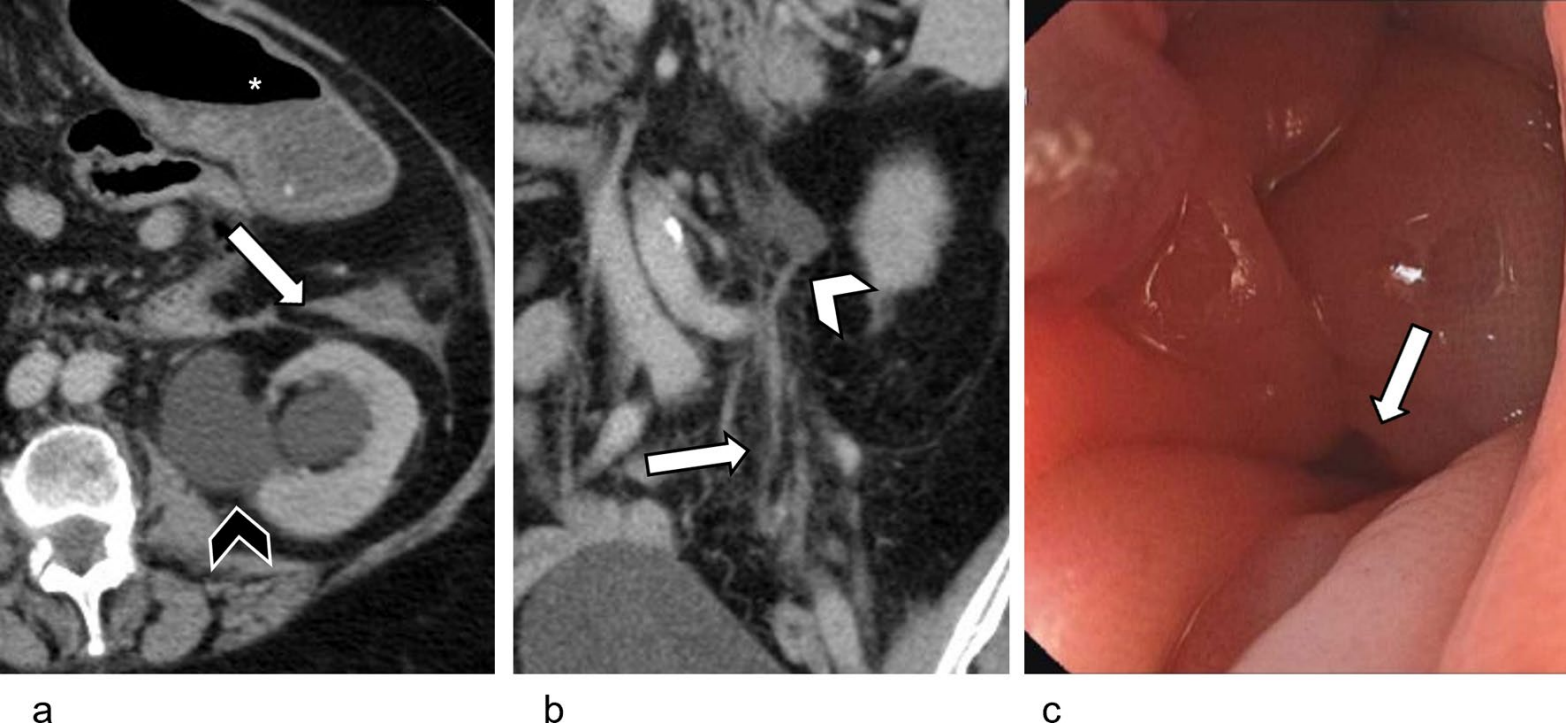
Metastatic retroperitoneal involvement in an 80-year-old female, who presented with symptoms of bowel obstruction. She had declined surgical treatment for Stage 2B ILC a year earlier and was on letrozole. **a** Axial contrast-enhanced CT image shows a stricture of the descending colon, with a tethered, tapered appearance at its medial aspect (white arrow). The upstream transverse colon (*) is dilated. Left-sided hydronephrosis is also seen (black arrowhead). **b** Coronal contrast-enhanced CT image shows tiny metastatic nodules and stranding in the left side of the retroperitoneum (white arrow). The transition points of the left hydronephrosis (white arrowhead) and descending colon (not pictured) are within this nodular area. **c** Colonoscopy confirms a stricture of the descending colon (white arrow). The mucosa appeared normal on colonoscopy, and this was initially thought to represent a benign stricture. Subsequent open laparotomy confirmed disseminated metastatic disease in the retroperitoneum

When confluent, ILC peritoneal metastases appear as omental caking [8]. Metastatic retroperitoneal disease progresses to retroperitoneal fibrosis, causing ureteral obstruction and hydronephrosis [40]. Extension to the bowel serosa causes stricture formation, with sparing of the mucosa, mimicking a benign stricture on colonoscopy (Fig. 9).

**Fig. 10:**
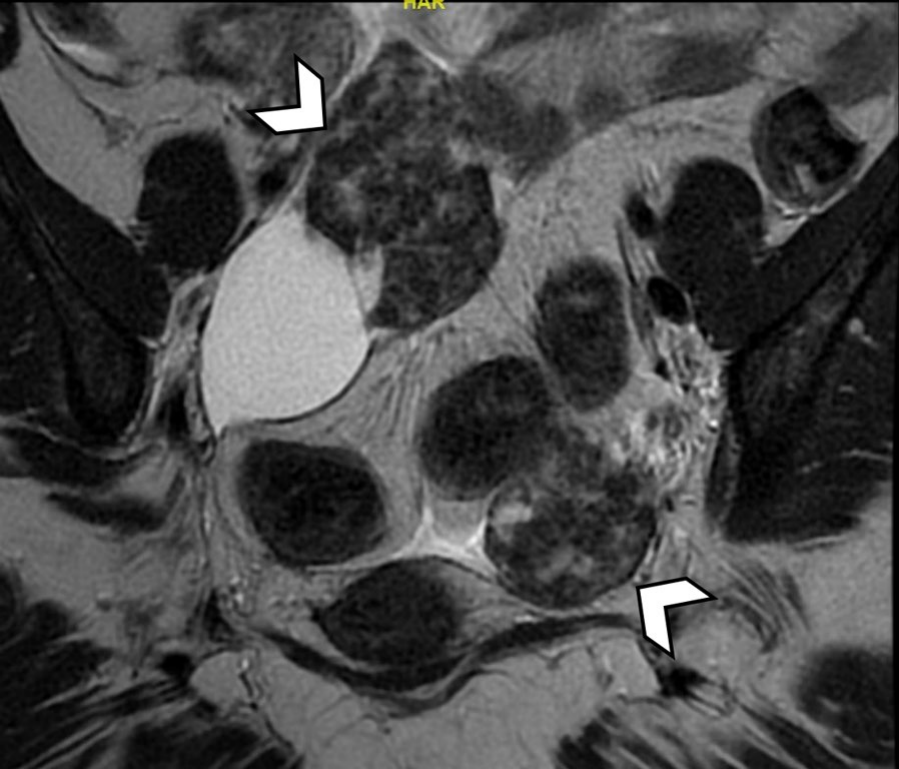
Bilateral solid ovarian masses first detected on staging CT in a 54-year-old female with newly diagnosed ILC. Coronal T2-weighted MR image of the pelvis shows bilateral, solid, ovarian masses with heterogeneous low T2-weighted signal (arrowheads), suggestive of fibrous, desmoplastic components. There is an ovarian cyst adjacent to the right ovarian mass. The solid appearance of the masses and low T2-weighted signal favor Krukenberg tumors over primary ovarian malignancy. Metastatic ILC involvement was confirmed following hysterectomy and bilateral salpingo-oophorectomy

When peritoneal nodules and ovarian masses are seen in the abdomen, it can be challenging to distinguish metastatic breast cancer from metastatic ovarian cancer [8]. This problem is compounded by the fact that ILC has a high incidence of metastasizing to the ovaries (up to 13%) [5, 29] and some breast cancer patients are at increased risk of developing primary ovarian cancer, such as patients with BRCA1 and BRCA2 mutations [41]. Bilateral solid ovarian tumors or Krukenberg tumors (Fig. 10) favor metastatic breast cancer infiltration of the ovaries, as primary ovarian malignancy tends to have a mixture of solid and cystic components [42, 43]. In a patient with known ILC and imaging findings of peritoneal nodules and solid ovarian masses, metastatic ILC and metastatic ovarian cancer are important differentials for the radiologist to raise.

## Conclusion

Evaluating patients with metastatic ILC in the abdomen is challenging. Due to its infiltrative pattern of spread, imaging findings could be subtle and easily missed until the disease is extensive.

Close discussion with clinicians and awareness of these imaging pitfalls will enable the radiologist to assess patients with a history of ILC with a high index of suspicion. This can direct immunohistochemical staining for correct diagnosis and provide the greatest value in treatment planning.

## Data Availability

Not applicable.

## Abbreviations

18F-FDG PET/CT: 18F-fluorodeoxyglucose positron emission tomography/ computed tomography
CEDM: Contrast-enhanced digital mammography
CT: Computed tomography
DBT: Digital breast tomosynthesis
EUS: Endoscopic ultrasound
GI: Gastrointestinal
IDC: Invasive ductal carcinoma
ILC: Invasive lobular carcinoma
MRE: MRI elastography
MRI: Magnetic resonance imaging
NST: Invasive carcinoma of no special type
SUV: Standardized uptake value
